# Supplementary material for: C-type lectin receptor 2d forms homodimers and heterodimers with TLR2 to negatively regulate IRF5-mediated antifungal immunity
Source: Nat Commun. 2023 Oct 23;14:6718. doi: 10.1038/s41467-023-42216-3 (PMC10593818; doi:10.1038/s41467-023-42216-3)
Supplement: Supplementary file 1 — Supplementary Information [file 41467_2023_42216_MOESM1_ESM.pdf]

Supplemental Information

C-type lectin receptor 2d forms homodimers and heterodimers with TLR2 to negatively regulate IRF5-mediated antifungal immunity

Fan Li [1,2,3,†], Hui Wang[2,3,†], Yan-Qi Li[2,3], Yebo Gu[1*], Xin-Ming Jia[2,3*]

[1]Department of Stomatology, Shanghai Tenth People's Hospital, School of Medicine, Tongji University, Shanghai 200072, China.

[2]Clinical Medicine Scientific and Technical Innovation Center, Shanghai Tenth People's Hospital, Tongji University School of Medicine, Shanghai 200072, China.

[3]Key Laboratory of Pathogen-Host Interactions of the Ministry of Education of China, Tongji University, Shanghai 200092, China.

[†]These authors contributed equally.

Supplemental Figures

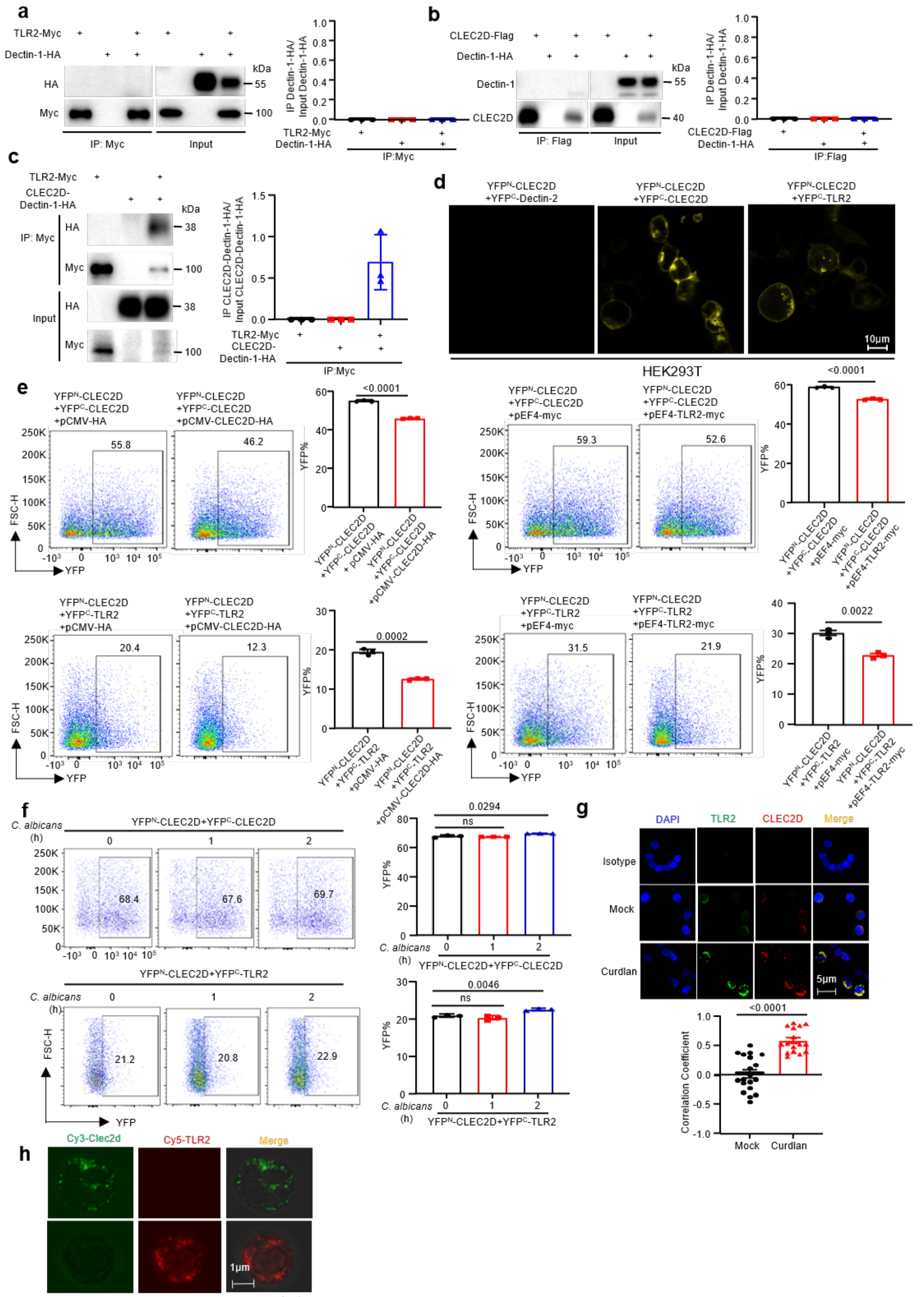

Supplementary Figure 1. Heterodimerization of CLEC2D and TLR2. (a-b) Left panel: Association of human Dectin-1with human TLR2 **(a)** and human CLEC2D **(b)** in HEK293T cells transfected with indicated proteins. Right panel: Quantitative analysis of IP Dectin-1-HA normalized to input Dectin-1-HA**(a-b). (c)** Left panel:

Immunoblotting analysis of interaction of the chimera of hCLEC2D (extracellular domain,60-191aa) and hDectin-1 (intracellular domain, 1-65aa) or hTLR2-Myc as indicated. Right panel: Quantitative analysis of IP CLEC2D-Dectin-1-HA normalized to input CLEC2D-Dectin-1-HA. **(d)** Bimolecular fluorescence complementation (BiFC) assay for detecting dimerization of YFP^N-CLEC2D with YFP^C-Dectin-2, YFP^C-CLEC2D, YFP^C-TLR2 by Immunofluorescence microscopy. Scar bars =10μm. **(e)** HEK293T cells were transfected with expression vectors in different combinations or together with plasmids expressing HA-tagged CLEC2D or Myc-tagged TLR2 and then examined by flow cytometry. **(f)** Flow cytometry assay for detecting dimerization of YFP^N-CLEC2D with YFP^C-CLEC2D and YFP^C-TLR2 in HEK 293T cells stimulated with *C. albicans* for indicated time. **(g)** Representative immunofluorescent staining assay of co-localization of CLEC2D (anti-CLEC2D Ab, red) and TLR2 (anti-TLR2 Ab, green) in Human PBMC (1×106 cells/well) stimulated with curdlan (20μg/well) for 60 min, Nuclear was stained with DAPI (2μg/ml) (bule). Pearson's correlation coefficients (PCCs) were used to quantify the co-localization of CLEC2D and TLR2. Scar bars = 10μm. **(h)** Immunofluorescent images of Cy3 conjugated anti-CLEC2D antibody only and Cy5 conjugated anti-TLR2 antibody only staining to human PBMCs, respectively. Scar bars =1μm.ns, no significance. Data were presented as mean ± SEM; n=3**(a-c, e-f),** n=19**(g, mock group), n=16(g, Curdlan group)** biologically independent samples. Data were analyzed by unpaired two-sided Student's *t*-test in **a-c** and **e-g**. Source data are provided as a Source Data file.

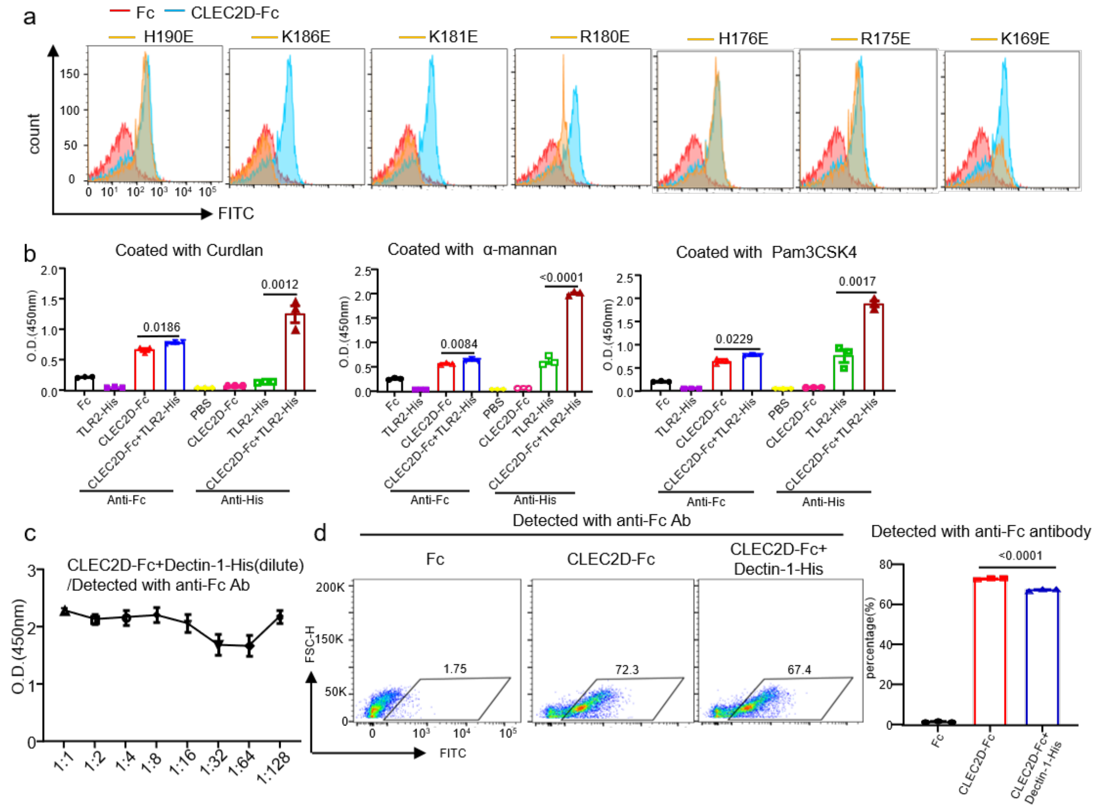

Supplementary Figure 2. CLEC2D homodimers or heterodimers have high affinity to β-glucans, α-mannan or Pam3CSK4. (a) Flow cytometry assay of the binding of *C. albicans* yeast cells with the indicated soluble amino acid mutated proteins of CLEC2D-Fc. **(b)** ELISA results for binding assays of TLR2-His, CLEC2D-Fc or a combination of the two proteins (TLR2-His+CLEC2D-Fc) with curdlan, α-mannan and Pam3CSK4(1μg/well). **(c)** ELISA results for the competitive binding of multiple diluted Dectin-1-His with CLEC2D-Fc to plated-coated curdlan (1μg/well), which was detected with anti-Fc antibody. **(d)** Flow cytometry assay of the competitive binding of Dectin-1-His with CLEC2D-Fc to *C. albicans* yeast cells, which was detected with anti-Fc antibody. Data were presented as mean ± SEM; n=3**(b-d)** biologically independent samples. Data were analyzed by unpaired two-sided Student's *t*-test in **b-d**. Source data are provided as a Source Data file.

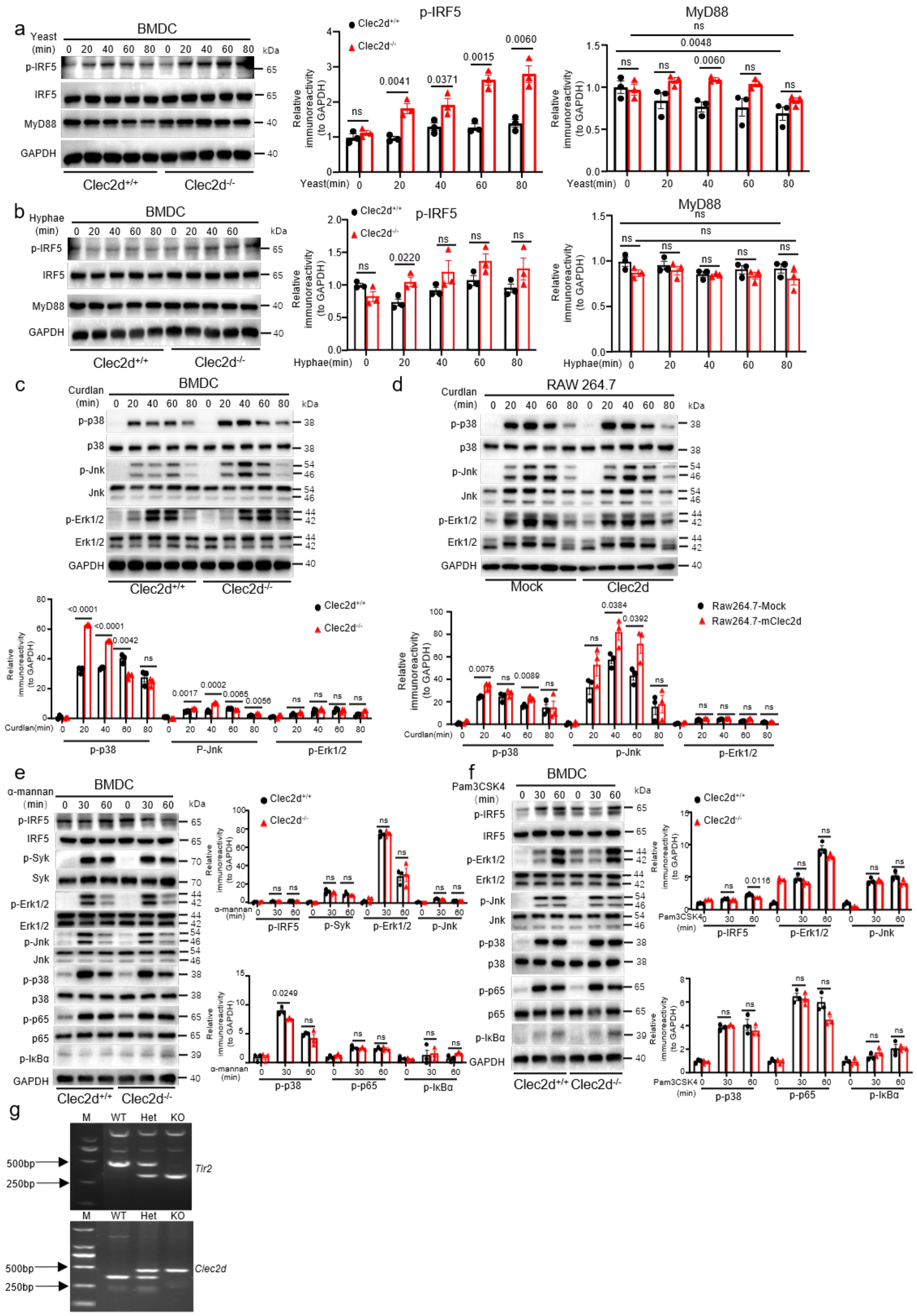

Supplementary Figure 3. Homo- and heterodimeric CLEC2D have no influence on β-glucan, α-mannan or Pam3CSK4-induced MAPKs signaling pathway activation. (a-b) Left panel: Immunoblotting analysis of IRF5 phosphorylation and MyD88 degradation in wild-type and *Clec2d*-deficient BMDCs stimulated with yeast**(a)** and hyphae**(b)** for indicated time. Middle panel: Quantitative analysis of p-IRF5

normalized to GAPDH**(a-b)**. Right panel: Quantitative analysis of MyD88 normalized to GAPDH**(a-b)**. **(c)**Upper panel: Immunoblotting analysis of curdlan induced phosphorylation of indicated proteins in BMDCs isolated from wild-type or *Clec2d* deficient mice. Lower panel: Quantitative analysis of p-p38, p-p65 and p-Erk1/2 normalized to GAPDH. **(d)** Upper panel: Immunoblotting analysis of curdlan induced phosphorylation of indicated proteins in RAW264.7 cells stably expressing mClec2d, or a control vector (mock) for indicated time. Lower panel: Quantitative analysis of p-p38, p-p65 and p-Erk1/2 normalized to GAPDH. **(e-f)** Immunoblotting and quantification analysis of α-mannan**(e)** or Pam3CSK4**(f)** induced phosphorylation of indicated proteins in BMDCs isolated from wild-type or *Clec2d* deficient mice. **(g)** Agarose gel images showing PCR products of genome of $Clec2d^{-/-}$ and $Clec2d^{-/-}TLR2^{-/-}$ mice. TLR2: wild type:499bp; Heterozygote:499bp and 334bp; knock out: 334bp. Clec2d: wild type:386bp; Heterozygote:486bp and 386bp; knock out: 486bp. Data were presented as mean ± SEM; n=3**(a-f)** biologically independent samples. Data were analyzed by one-way ANOVA adjusted for multiple comparisons in **a-f**. M: marker. WT: wild type. Het: heterozygote. KO: knockout. Source data are provided as a Source Data file.

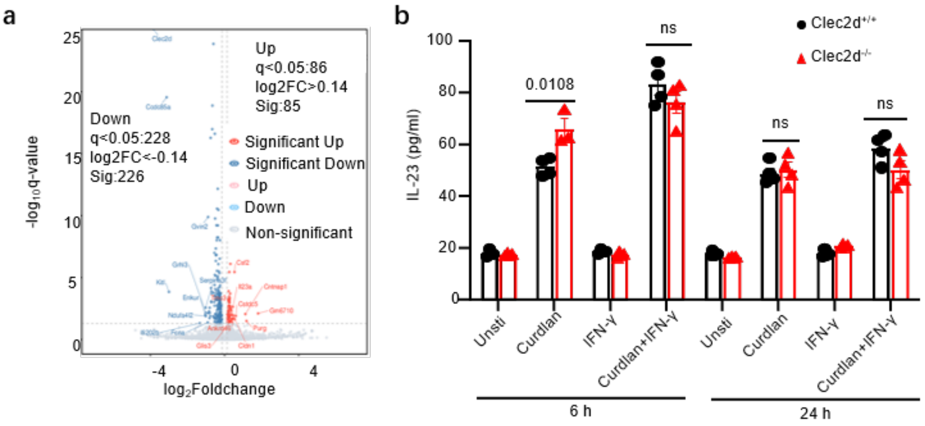

Supplementary Figure 4. The differentially expressed genes (DEGs) regulated by CLEC2D. (a) 226 downregulated (blue) and 85 upregulated genes (red) in BMDCs from *Clec2d*$^{+/+}$ and *Clec2d*$^{-/-}$ mice, which were generated by GM-CSF (20ng/ml) for 8 days and then treated with curdlan (10μg/well) for 3h, the volcano plot showed differentially expressed genes based on absolute fold change >1.1 and FDR <0.05. **(b)** ELISA results of IL23 production in the supernatants of wild-type and *Clec2d*-deficient BMDCs, which were stimulated with Curdlan (5μg/well). ns, no significance. Data were presented as mean ± SEM; n=4**(b)** biologically independent samples. Data were analyzed by one-way ANOVA adjusted for multiple comparisons in **b**. Source data are provided as a Source Data file.

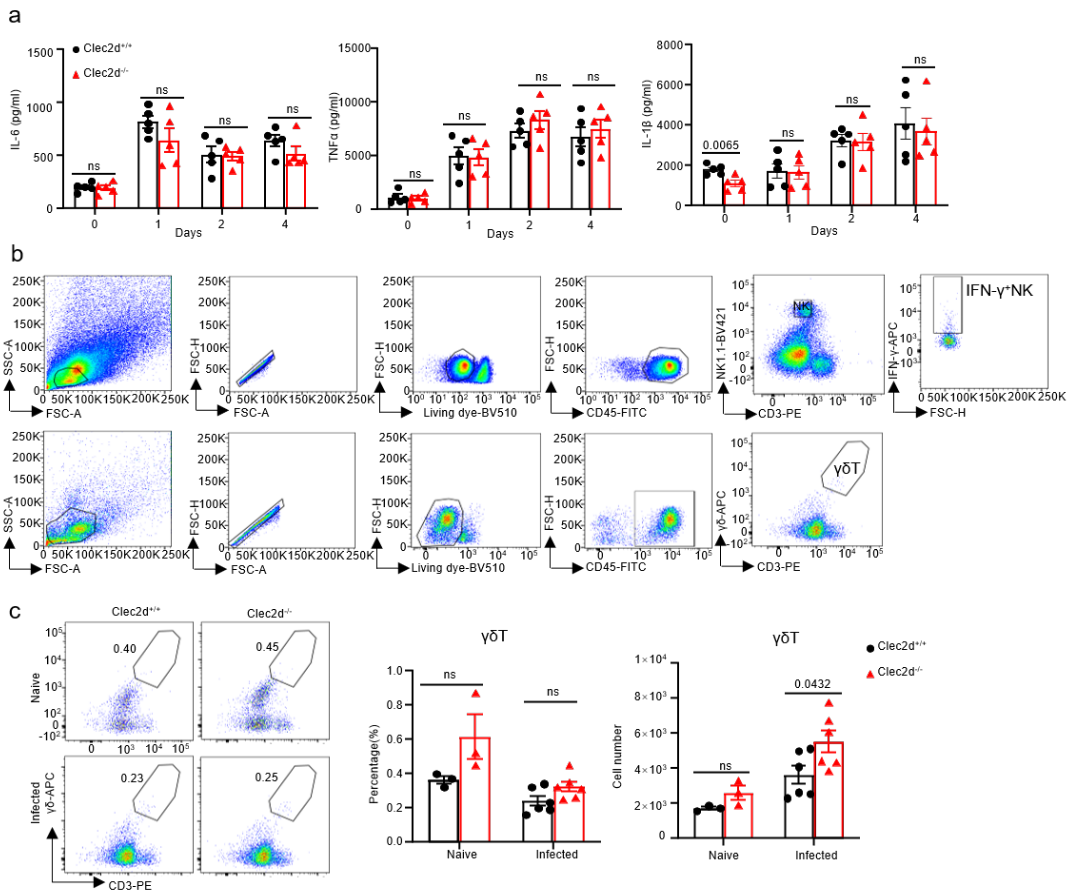

Supplementary Figure 5. ***Clec2d*** **deficiency induces activation of NK cells, but not γδT cells, to exert resistance effects.** **(a)**Production of IL-6, TNF-α and IL-1β in kidney tissue homogenate of wild type and *Clec2d*-deficient mice infected with 2×10^5 CFU of *C. albicans* at the indicated times. **(b)** Gating strategy of $CD45^+CD3^-$ $NK1.1^+$NK cells, $CD45^+CD3^-NK1.1^+IFN\text{-}\gamma^+$ NK cells and $CD45^+CD3^+\gamma\delta^+$ T cells. **(c)** Representative flow charts of the percentage and absolute number of γδT cells in kidney from *C. albicans* (2×10^5 CFU) infected wild type or *Clec2d*-deficient mice on day 1. ns, no significance. Data were presented as mean ± SEM; n=5 (**a**), n=3(**c** naive group), n=6(**c** infected group) biologically independent samples. Data were analyzed by one-way ANOVA adjusted for multiple comparisons in **a, c**. Source data are provided as a Source Data file.

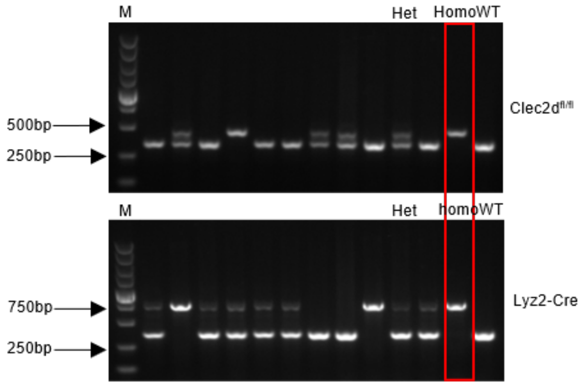

Supplementary Figure 6. Genotyping of mice with *Lyz2*$^{+}$ myeloid cell-specific deficiency *Clec2d*. Agarose gel images showing PCR products of genome of *Clec2d*$^{fl/fl}$ and *Clec2d*$^{fl/fl}$ *Lyz2*$^{cre/+}$ mice. *Clec2d*$^{fl/fl}$: wild-type:325bp; targeted:427bp. Lyz2-Cre: wild type:350bp; targeted:700bp. M: Marker. Het: heterozygote. Homo: homozygote. WT: wild type. Source data are provided as a Source Data file.

Supplemental Tables

Supplemental Table 1 Primer sequences used in this study

Gene	Sequences 5'-3'
**Primers for mice genotyping**	
Lyz2-Cre-Mutant	CCCAGAAATGCCAGATTACG
Lyz2-Cre-Common	CTTGGGCTGCCAGAATTTCTC
Lyz2-Cre-Wild type	TTACAGTCGGCCAGGCTGAC
Clec2d$^{fl/fl}$-F	ACTGACTAGGTTCTCTGAGACTGATCC
Clec2d$^{fl/fl}$-R	GACTGAGAATAACACTGAGTGCCAAG
Clec2d$^{-/-}$-F1	TAACTCCTGTCCTCTAG
Clec2d$^{-/-}$-R1	ATATGGGAGCCACAACTG
Clec2d$^{-/-}$-R2	GAGAACACCAATCTCAAAAAAC
TLR2$^{-/-}$Common	CTTCCTGAATTTGTCCAGTACA
TLR2$^{-/-}$Mutant	GGGCCAGCTCATTCCTCCCAC
TLR2$^{-/-}$Wild type	ACGAGCAAGATCAACAGGAGA
**Primers for qPCR**	
mouse *IL-12a* F	CTGTGCCTTGGTAGCATCTATG
mouse *IL-12a* R	GCAGAGTCTCGCCATTATGATTC
mouse *IL-12b* F	TGGTTTGCCATCGTTTTGCTG
mouse *IL-12b* R	ACAGGTGAGGTTCACTGTTTCT
mouse *IL-23a* F	ATGCTGGATTGCAGAGCAGTA
mouse *IL-23a* R	ACGGGGCACATTATTTTTAGTCT
mouse *Gapdh* F	AGGTCGGTGTGAACGGATTTG
mouse *Gapdh* R	TGTAGACCATGTAGTTGAGGTCA
**Primers for CHIP-qPCR**	
mouse IL-12a promotor F	CGTTGAAATCAGCCCTCAGC
mouse IL-12a promotor R	GCCAGTGATGAGTACAGCCT
mouse IL-12b promotor F	TTCATGTACCCCTTCAAGCCT
mouse IL-12b promotor R	AACCCAGCTCTCCACTTTGA
**Primers for plasmids construction**	
human CLEC2D-Fc-F	AGTCTTGCACTTGTCACGAATTCGAGAGC TAACTGCCATCAAGA
human CLEC2D-Fc-R	GCATGTGTGAGTTTTGTCAGATCTGACAT GTATATCTGATTTGG
human pEF4-TLR2-myc-F	GTAAGCTTGGTACCGAGCTCGATGCCACA TACTTTGTGGATG
human pEF4-TLR2-myc-R	TTCGAACCGCGGGCCCTCTAGGGACTTTAT CGCAGCTCTCAG
human pCMV-IRF5-HA-F	CTTATGGCCATGGAGGCCCGAAACCAGTC CATCCCAGTGGCT
human pCMV-IRF5-HA-R	CGCGGCCGCGGTACCTCGAGATTATTGCAT GCCAGCTGGGTG

human pLV-CLEC2D-Flag-F	CTGAGCTCCTTAAGGTTAACTATGCATGAC AGTAACAATGT
human pLV-CLEC2D-Flag-R	AGAGGGGCCCCGGGAATATTGCTACTTG TCGTCATCGTCTTTGTAG
human pLV-Dectin-1-HA- F	CTGAGCTCCTTAAGGTTAACTATGGAAT ATCATCCTGATTTAG
human pLV-Dectin-1-HA- R	AGAGGGGCCCCGGGAATATTGTTAAGC GTAATCTGGAACATC
human pLV-Myd88-His-F	CTGAGCTCCTTAAGGTTAACTATGGCTG CAGGAGGTCCCGGCGC
human pLV-Myd88-His-R	AGAGGGGCCCCGGGAATATTGCTAATG GTGATGGTGATGATG
human CLEC2DΔcyto 39-191-F	CTTATGGCCATGGAGGCCCGATTTTTCTTAATC ATGTTTC
human CLEC2DΔcyto 39-191-R	CGCGGCCGCGGTACCTCGAGACTAGACATGTAT ATCTG
human CLEC2DΔTM 1-38-F	CTTATGGCCATGGAGGCCCGACATGACAGTAAC AATG
human CLEC2DΔTM 1-38-R	TAAGCGCCAAATTAAGG
human CLEC2DΔTM 60-191-F	CTTAATTTGGCGCTTAAGAGCTAACTGCCATC
human CLEC2DΔET 1-59-R	CGCGGCCGCGGTACCTCGAGACTATATTGCGCT TAAAGCAGC
human TLR2-ΔET-F	GTAAGCTTGGTACCGAGCTCGATGGCACTGGTG TCTGGCATGTGCTG
human TLR2-ΔET-R	GTTCGAACCGCGGGCCCTCTAGGGACTTTATCG CAGCTCTCAG
human TLR2-ΔTM-ΔCYto-F	GTAAGCTTGGTACCGAGCTCGATGCCACATACT TTGTGGATGGTGTGGG
human TLR2-ΔTM-ΔCYto-R	GTTCGAACCGCGGGCCCTCTAGTGTCCTGTGAC ATTCCGACACCGAG
