# Supplementary material for: C-type lectin receptor 2d forms homodimers and heterodimers with TLR2 to negatively regulate IRF5-mediated antifungal immunity
Source: Nat Commun. 2023 Oct 23;14:6718. doi: 10.1038/s41467-023-42216-3 (PMC10593818; doi:10.1038/s41467-023-42216-3)
Supplement: Supplementary file 3 — Reporting Summary [file 41467_2023_42216_MOESM3_ESM.pdf]

nature portfolio

Corresponding author(s): Yebo Gu and Xin-Ming Jia

Last updated by author(s): Sep 27, 2023

Reporting Summary

Nature Portfolio wishes to improve the reproducibility of the work that we publish. This form provides structure for consistency and transparency in reporting. For further information on Nature Portfolio policies, see our Editorial Policies and the Editorial Policy Checklist.

Statistics

For all statistical analyses, confirm that the following items are present in the figure legend, table legend, main text, or Methods section.

n/a	Confirmed	
☐	☒	The exact sample size (n) for each experimental group/condition, given as a discrete number and unit of measurement
☐	☒	A statement on whether measurements were taken from distinct samples or whether the same sample was measured repeatedly
☐	☒	The statistical test(s) used AND whether they are one- or two-sided<br>*Only common tests should be described solely by name; describe more complex techniques in the Methods section.*
☐	☒	A description of all covariates tested
☐	☒	A description of any assumptions or corrections, such as tests of normality and adjustment for multiple comparisons
☐	☒	A full description of the statistical parameters including central tendency (e.g. means) or other basic estimates (e.g. regression coefficient) AND variation (e.g. standard deviation) or associated estimates of uncertainty (e.g. confidence intervals)
☐	☒	For null hypothesis testing, the test statistic (e.g. F, t, r) with confidence intervals, effect sizes, degrees of freedom and P value noted<br>*Give P values as exact values whenever suitable.*
☒	☐	For Bayesian analysis, information on the choice of priors and Markov chain Monte Carlo settings
☒	☐	For hierarchical and complex designs, identification of the appropriate level for tests and full reporting of outcomes
☐	☒	Estimates of effect sizes (e.g. Cohen's d, Pearson's r), indicating how they were calculated

Our web collection on statistics for biologists contains articles on many of the points above.

Software and code

Policy information about availability of computer code

Data collection	Flow cytometry data was collected by FACSCelesta(BD Biosciences) using BD DIVA8.0.1 software (BD Biosciences).<br>qPCR was done by Roche LightCycler96 sequencing detection system.<br>Western blot gels were obtained by Fully Automatic chemiluminescence/fluorescence image analysis System(Tanon 5200s).<br>Immunofluorescence images were obtained by Laser confocal microscope using ZEN2.6(black edition and blue edition)(Zeiss LSM880).<br>ELISA data was collected by Multiscan FC(Thermo Fisher).<br>The libraries were sequenced on the Illumina sequencing platform(HiSeqTM 2500 or Illumina HiSeq X Ten).
Data analysis	Flow cytometry data was analyzed by Flowjo10.<br>Data analysis and plotted were using GraphPad Prism8.<br>Wester blots gels were quantified with ImageJ(version 1.8.0).

For manuscripts utilizing custom algorithms or software that are central to the research but not yet described in published literature, software must be made available to editors and reviewers. We strongly encourage code deposition in a community repository (e.g. GitHub). See the Nature Portfolio guidelines for submitting code & software for further information.

Data

Policy information about availability of data

All manuscripts must include a data availability statement. This statement should provide the following information, where applicable:

- Accession codes, unique identifiers, or web links for publicly available datasets
- A description of any restrictions on data availability
- For clinical datasets or third party data, please ensure that the statement adheres to our policy

The data supporting the findings from this study are available within the article file and its supplementary information/source data file. Any other raw data or non-commercial material used in this study are available from the corresponding author upon reasonable request. Source data are provided as a Source Data file.

Research involving human participants, their data, or biological material

Policy information about studies with human participants or human data. See also policy information about sex, gender (identity/presentation), and sexual orientation and race, ethnicity and racism.

Reporting on sex and gender	*Use the terms sex (biological attribute) and gender (shaped by social and cultural circumstances) carefully in order to avoid confusing both terms. Indicate if findings apply to only one sex or gender; describe whether sex and gender were considered in study design; whether sex and/or gender was determined based on self-reporting or assigned and methods used. Provide in the source data disaggregated sex and gender data, where this information has been collected, and if consent has been obtained for sharing of individual-level data; provide overall numbers in this Reporting Summary. Please state if this information has not been collected. Report sex- and gender-based analyses where performed, justify reasons for lack of sex- and gender-based analysis.*
Reporting on race, ethnicity, or other socially relevant groupings	*Please specify the socially constructed or socially relevant categorization variable(s) used in your manuscript and explain why they were used. Please note that such variables should not be used as proxies for other socially constructed/relevant variables (for example, race or ethnicity should not be used as a proxy for socioeconomic status). Provide clear definitions of the relevant terms used, how they were provided (by the participants/respondents, the researchers, or third parties), and the method(s) used to classify people into the different categories (e.g. self-report, census or administrative data, social media data, etc.) Please provide details about how you controlled for confounding variables in your analyses.*
Population characteristics	*Describe the covariate-relevant population characteristics of the human research participants (e.g. age, genotypic information, past and current diagnosis and treatment categories). If you filled out the behavioural & social sciences study design questions and have nothing to add here, write "See above."*
Recruitment	*Describe how participants were recruited. Outline any potential self-selection bias or other biases that may be present and how these are likely to impact results.*
Ethics oversight	*Identify the organization(s) that approved the study protocol.*

Note that full information on the approval of the study protocol must also be provided in the manuscript.

Field-specific reporting

Please select the one below that is the best fit for your research. If you are not sure, read the appropriate sections before making your selection.

☒ Life sciences ☐ Behavioural & social sciences ☐ Ecological, evolutionary & environmental sciences

For a reference copy of the document with all sections, see nature.com/documents/nr-reporting-summary-flat.pdf

Life sciences study design

All studies must disclose on these points even when the disclosure is negative.

Sample size	The minimum samples of each experiment was n=3, and up to n=12(in survival experiments). The exact n for each experiment was described in corresponding figure legends. Sample sizes were determined based on previous experiences and standards in the field. Low variability between the same type of samples, indicated as SEM, conforming that n=3-6 samples is sufficient to observe statistically significant differences between relevant groups. Whereas for survival rate experiments, at least n=10 samples were used as figure legends indicated.
Data exclusions	No data was excluded from analysis.
Replication	At least three independent were performed for each experiments, each panel presented the representative data. All reported data were reproduced reliably.
Randomization	For animal models, all mice (WT, Clec2d-/-, Clec2dfl/fl, Clec2dfl/flLyz2Cre/+,TLR2-/-, Clec2d-/-TLR2-/-) used in this study were divided into different treatment groups randomly. For cell culture, like BMDC in vitro generation, ELISA assay, RT-qPCR assay and Western blot, cells were divided into each plates and assigned into different treatments groups randomly.

Blinding	All data acquisition and analysis in this study were performed in a blinded way.

Reporting for specific materials, systems and methods

We require information from authors about some types of materials, experimental systems and methods used in many studies. Here, indicate whether each material, system or method listed is relevant to your study. If you are not sure if a list item applies to your research, read the appropriate section before selecting a response.

Materials & experimental systems

n/a	Involved in the study
☐	☒ Antibodies
☐	☒ Eukaryotic cell lines
☒	☐ Palaeontology and archaeology
☐	☒ Animals and other organisms
☒	☐ Clinical data
☒	☐ Dual use research of concern
☒	☐ Plants

Methods

n/a	Involved in the study
☒	☐ ChIP-seq
☐	☒ Flow cytometry
☒	☐ MRI-based neuroimaging

Antibodies

Antibodies used

Antibodies used in this paper:
For flow cytometry
FITC-anti mouse CD45(I3/2.3) Biolegend 147710;1:100
PE- anti mouse CD3(17A2) Biolegend 100205;1:100
BV421-anti mouse NK1.1(PK136) Biolegend 108741;1:100
APC-anti mouse γδ(GL3) Biolegend 118116;1:100
PerCP-Cy5.5-anti mouse CD11b (M1/70) BD Pharmingen 561114;1:100
BV421-anti mouse Ly-6G (1A8) Biolegend 127628;1:100
BV421-anti mouse CD19(6D5) Biolegend 115549;1:100
BV421-anti mouse CD90.2(30-H12) Biolegend 105341;1:100
APC-anti mouse IFNγ (XMG1.2) Biolegend 505810;1:100
FITC-anti-human Fc fragment Jackson Immuno Research 109-007-008;1:100
PE-anti mouseCD369(Dectin-1/CLEC7A)(RH1) Biolegend;1:20
Fixable Viability Stain 510 BD Pharmingen 564406;1:200

For microscopy
Alexa Flour 594-labeled donkey anti-mouse IgG abcam ab150116; 1:200
FITC anti-human CD282 (TLR2) Antibody Biolegend 309706;1:100
Anti-1,3 beta glucan(2G8) abcam Ab233743:1:100
Anti-human Clec2d Jia's lab(Clone 2260CT10.1.3.2);1:100
Dectin-1/CLEC7A protein, Human (HEK293, Fc) MedChemExpress HY-P70101;5μg/mL
Calcofluor White sigma-aldrich 18909;50 μg/mL
FITC ConA sigma-aldrich C7642; 5μg/mL
DAPI Beyotime Biotech C1002;2μg/mL

For Western blot
phospho-p38 Cell Signaling Technology 4511S;1:1000
p38 Cell Signaling Technology 8690S;1:1000
phospho-ERK Cell Signaling Technology 4370S;1:1000
ERK Cell Signaling Technology 4695S;1:1000
GAPDH Cell Signaling Technology 5174S;1:1000
p65 Cell Signaling Technology 8242S;1:1000
phospho-p65 Cell Signaling Technology 3033S;1:1000
syk Cell Signaling Technology 13198S;1:1000
phospho-syk Cell Signaling Technology 2710S;1:1000
Jnk Cell Signaling Technology 9252S;1:1000
phospho-Jnk Cell Signaling Technology 4668S;1:1000
IRF5 abcam ab181553;1:1000
phospho-IRF5 Thermo Fisher PA5-64760;1:1000
mClec2d R&D AF3376-SP;1:1000
PCNA Cell Signaling Technology 13110S;1:1000
MyD88 Cell Signaling Technology 4283S;1:1000
phospho-IκBα Cell Signaling Technology 9246S;1:1000
Myc-Tag Abmart M20002L;1:1000
Flag-Tag Abmart M20008L;1:1000
HA-Tag Cell Signaling Technology 3724S;1:1000
TLR2 Cell Signaling Technology 13744S;1:1000
human Fc fragment Jackson Immuno Research 109-005-008;1:1000

Goat anti-mouse IgG-HRP Abmart M21001L;1:10000
Goat anti-rabbit IgG-HRP Abmart M21002L;1:10000
Donkey anti-goat IgG-HRP YEASEN 34301ES60;1:10000

For Co-immunoprecipitation
TLR2 Abcam ab209217;2μg/sample
TUBE 1 (Magnetic Beads) LifeSensors UM401M;50μL/sample
Anti-Flag Magnetic Beads MedChemExpress HY-K0207; 15μL/sample
PierceTM Protein A/G Magnetic Beads Thermo Fisher 88803;10μl/sample

For neutralization
Anti-mouse IL-12p70(R2-9A5) BioX Cell BE0233;250μg/mouse
Rat IgG2b isotype control (LTF-2) BioX Cell BE0090;250μg/mouse
Anti-mouse NK1.1 (PK136) BioX Cell BE0036; 350μg/mouse
Mouse IgG2a isotype control BioX Cell (C1.18.4); 350μg/mouse

For ELISA assay
IL-12p40 Thermo Fisher 88-7120-88
IL-12p70 Thermo Fisher 88-7121-88
IL-6 Thermo Fisher 88-7064-88
IFN-γ Thermo Fisher 88-7314-88
TNF-α Thermo Fisher 88-7324-77
IL-23 Thermo Fisher 88-7230-88

For FRET
Cy5 conjugated anti-human TLR2 Bioss,bs-10472R-Cy5;1:10
Cy3 conjugated anti-human CLEC2D Bioss, bs-2683R-Cy3;1:10

Validation

All antibodies were validated before use. This involve appropriate negative and positive control.
For flow cytometry antibodies, we relied on the manufacturer's validation which were used in many labs and published papers, and we performed isotype control when we first use, including FITC-anti mouse CD45(I3/2.3) Biolegend, PE- anti mouse CD3(17A2) Biolegend, BV421-anti mouse NK1.1(PK136) Biolegend, APC-anti mouse γδ(GL3) Biolegend, PerCP-Cy5.5-anti mouse CD11b (M1/70) BD Pharmingen, BV421-anti mouse Ly-6G (1A8) Biolegend, BV421-anti mouse CD19(6D5) Biolegend, BV421-anti mouse CD90.2(30-H12) Biolegend, APC-anti mouse IFNγ (XMG1.2) Biolegend, FITC-anti-human Fc fragment Jackson Immuno Research, PE-anti mouse CD369(Dectin-1/CLEC7A)(RH1) Biolegend.

For microscope
Detailed validation from manufacturer's data sheets:
-Alexa Flour 594-labeled donkey anti-mouse IgG abcam ab150116
IHC-Fr, ICC/IF were validated in published papers, the recommended concentration is 1/200-1/1000.
-FITC anti-human CD282 (TLR2) Antibody Biolegend 309706
FC were validated in published papers, and IF were validated by us, the concentration is 1:100.
-Anti-1,3 beta glucan(2G8) abcam Ab233743
ELISA were validated in published papers, and IF were validated by us, the concentration is 1:100.
-Anti-human Clec2d Jia's lab(Clone 2260CT10.1.3.2)
IF were validated when we first use, and the concentration is 1:100.
-Dectin-1/CLEC7A protein, Human (HEK293, Fc) MedChemExpress HY-P70101
IF were were validated when we first use, and the concentration is 5μg/mL.
-Calcofluor White sigma-aldrich 18909;50 μg/mL
IF were were validated in published papers, and the concentration is 50μg/mL.
-FITC ConA sigma-aldrich C7642; 5μg/mL
IF were were validated in published papers, and the concentration is 5μg/mL.
-DAPI Beyotime Biotech C1002;2μg/mL
IF were were validated in published papers, and the concentration is 2μg/mL.

For western blot
Detailed validation from manufacturer's data sheets:
-phospho-p38 Cell Signaling Technology 4511S;
Western blot analysis of extracts from COS and 293 cells, untreated or UV-treated, using Phospho-p38 MAPK (Thr180/Tyr182) (D3F9) XP® Rabbit mAb.
-p38 Cell Signaling Technology 8690S;
Western blot analysis of extracts from various cell lines using p38 MAPK (D13E1) XP® Rabbit mAb.
-phospho-ERK Cell Signaling Technology 4370S;
Western blot analysis of extracts from COS cells, untreated or treated with either U0126 #9903 (10 μM for 1h) or TPA #4174 (200 nM for 10 m), using Phospho-p44/42 MAPK (Erk1/2) (Thr202/Tyr204) (D13.14.4E) XP® Rabbit mAb #4370 .
-ERK Cell Signaling Technology 4695S;
Western blot analysis of extracts from HeLa, NIH/3T3 and C6 cells, using p44/42 MAPK (Erk1/2) (137F5) Rabbit mAb.
-GAPDH Cell Signaling Technology 5174S;
Western blot analysis of extracts from various cell lines using GAPDH (D16H11) XP® Rabbit mAb.

-p65 Cell Signaling Technology 8242S;
Western blot analysis of extracts from various cell lines using NF-κB p65 (D14E12) XP® Rabbit mAb.
-phospho-p65 Cell Signaling Technology 3033S;
Western blot analysis of extracts from HeLa and NIH/3T3 cells, untreated or TNF-α treated (#2169, 20 ng/ml for 5 minutes), using Phospho-NF-κB p65 (Ser536) (93H1) Rabbit mAb.
-syk Cell Signaling Technology 13198S;
Western blot analysis of extracts from various cell lines using Syk (D3Z1E) XP® Rabbit mAb.
-phospho-syk Cell Signaling Technology 2710S;
Western blot analysis of extracts from Ramos cells, untreated or treated with anti-IgM, using Phospho-Syk (Tyr525/526) (C87C1) Rabbit mAb
-Jnk Cell Signaling Technology 9252S;
Western blot analysis of extracts from 293 and SK-N-MC cells, untreated or UV-treated (40 J/m2), using Phospho-SAPK/JNK Antibody #9251.
-phospho-Jnk Cell Signaling Technology 4668S;
Western blot analysis of extracts from 293 cells, untreated or UV-treated, NIH/3T3 cells, untreated or UV-treated and C6 cells, untreated or anisomycin-treated, using Phospho-SAPK/JNK (Thr183/Tyr185) (81E11) Rabbit mAb.
-IRF5 abcam ab181553;
Suitable for: Flow Cyt (Intra), WB, IHC-P, ICC/IF, IP,knockout validated.
-phospho-IRF5 Thermo Fisher PA5-64760;
WB was validated in published paper.
-mClec2d R&D AF3376-SP;
WB was validated in published paper.
-PCNA Cell Signaling Technology 13110S;
Western blot analysis of extracts from various cell lines using PCNA (D3H8P) XP® Rabbit mAb.
-MyD88 Cell Signaling Technology 4283S;
Western blot analysis of extracts from Raji, A549, and THP-1 cells using MyD88 (D80F5) Rabbit mAb.
-phospho-IκBα Cell Signaling Technology 9246S;
Western blot analysis of extracts from NIH/3T3 cells, untreated or TNF-α-treated (#8902, 20 ng/ml) for 5 minutes, using Phospho-IκBα (Ser32/36) (5A5) Mouse mAb #9246.
-Myc-Tag Abmart M20002L;
WB and IP were validated in published paper.
-Flag-Tag Abmart M20008L;
WB and IP were validated in published paper.
-HA-Tag Cell Signaling Technology 3724S;
Western blot analysis of extracts from HeLa cells, untransfected or transfected with either HA-FoxO4 or HA-Akt3, using HA-Tag (C29F4) Rabbit mAb.
-TLR2 Cell Signaling Technology 13744S;
Western blot analysis of extracts from Raw 264.7 cells, mouse bone marrow-derived macrophages (BMDM), and mouse bone marrow-derived dendritic cells (BMDC) using Toll-like Receptor 2 (E1J2W) Rabbit mAb.
-human Fc fragment Jackson Immuno Research 109-005-008;
WB was validated by us using human IgG protein.
-Goat anti-mouse IgG-HRP Abmart M21001L;
WB was validated in published paper.
-Goat anti-rabbit IgG-HRP Abmart M21002L;
WB was validated in published paper.
-Donkey anti-goat IgG-HRP YEASEN 34301ES60;
WB was validated in published paper.

For Co-immunoprecipitation
Detailed validation from manufacturer's data sheets:
-TLR2 Abcam ab209217;
WB: Mouse and rat spleen tissue lysates; RAW 264.7 whole cell lysates untreated and treated with 1 μg/ml lipopolysaccharides for 6 hours . IP: RAW 264.7 treated with 1 μg/ml LPS for 6 hours whole cell lysate.
-TUBE 1 (Magnetic Beads) LifeSensors UM401M;
IP was validated in published paper.
-Anti-Flag Magnetic Beads MedChemExpress HY-K0207;
IP was validated in published paper.
-PierceTM Protein A/G Magnetic Beads Thermo Fisher 88803;
High capacity purification of IgG from rabbit and mouse serum was validated by manufacturer.

For neutralization
Detailed validation from manufacturer's data sheets:
-Anti-mouse IL-12p70(R2-9A5) BioX Cell BE0233;
Depletion efficiency was validated in published papers.
-Anti-mouse NK1.1 (PK136) BioX Cell BE0036;
Depletion efficiency was validated in published papers.

For ELISA assay
Detailed validation from manufacturer's data sheets, and all the antibodies used for ELISA assay have been validated in published papers, including IL-12p40 Thermo Fisher 88-7120-88, IL-12p70 Thermo Fisher 88-7121-88, IL-6 Thermo Fisher 88-7064-88, IFN-γ Thermo Fisher 88-7314-88, TNF-α Thermo Fisher 88-7324-77, IL-23 Thermo Fisher 88-7230-88.

For FRET
Detailed validation from manufacturer's data sheets:
-Cy5 conjugated anti-human TLR2 Bioss,bs-10472R-Cy5;
IF were validated in published papers, the recommended concentration is 1/50-1/200.

-Cy3 conjugated anti-human CLEC2D Bioss,bs-2683R-Cy3;
IF were validated in published papers, the recommended concentration is 1/50-1/200.

Eukaryotic cell lines

Policy information about cell lines and Sex and Gender in Research

Cell line source(s)	Raw264.7 and HEK293T cell lines are reserved in Jia's lab.
Authentication	These cell lines were passaged in laboratory and authenticate by sociobiological company.
Mycoplasma contamination	Raw264.7 and HEK293T cell lines were tested regularly by using GMyc-PCR Mycoplasma Test Kit(40601ES10,Yeasen, China) and has no Mycoplasma contamination.
Commonly misidentified lines (See ICLAC register)	No commonly misidentified cell lines were included in this study.

Animals and other research organisms

Policy information about studies involving animals; ARRIVE guidelines recommended for reporting animal research, and Sex and Gender in Research

Laboratory animals	All mice used in this study are 6-8 weeks. C57BL/6 mice were purchased from Shanghai SLAC animal laboratory,Clec2d-/- mice line was generated using CRISPR–Cas9 methods,Lyz2Cre and Clec2dfl/fl mice were purchased from GemPharmatech (Nanjing, China), Clec2dfl/fl mice were crossed to Lyz2Cre to obtain Lyz2Cre/+Clec2dfl/fl. TLR2 knockout mice were purchased from The Jackson Laboratory (USA). To obtain Clec2d-/-TLR2-/- mice, Clec2d-/- mice were crossbred with TLR2-/- mice.The animal housing environment was maintained under specific pathogen-free conditions with a 12-hour light and 12-hour dark cycle. All mice were provided with free access to food and water to ensure adequate nutrition and hydration. All animal studies were performed using sex-and age-matched female mice.
Wild animals	No wild animals were involved in this study.
Reporting on sex	Only female mice were involved in this study.
Field-collected samples	This study did not involve Field-collected samples.
Ethics oversight	All animal experiments were performed according to the protocol approved by the Animal Ethics Committee of Tongji University School of Medicine (protocol No. TJAA09021101). Studies of human PBMCs were approved by the Human Research Committee of Tongji University School of Medicine (protocol No. 2021TJDX019).

Note that full information on the approval of the study protocol must also be provided in the manuscript.

Flow Cytometry

Plots

Confirm that:

☒ The axis labels state the marker and fluorochrome used (e.g. CD4-FITC).

☒ The axis scales are clearly visible. Include numbers along axes only for bottom left plot of group (a 'group' is an analysis of identical markers).

☒ All plots are contour plots with outliers or pseudocolor plots.

☒ A numerical value for number of cells or percentage (with statistics) is provided.

Methodology

Sample preparation	Single-cell suspensions from kidneys were obtained by collagenase digestion. For enriching immunocytes in kidney, cells were centrifuged by using 40% and 80% Percoll gradients and harvested cell layer between them. Cells were stained with fluorochrome-conjugated antibodies, according to the manufacturer's protocols. Flow cytometry was performed with a BD FACSFertasa flow cytometry system (BD Biosciences, San Jose, Calif), and data were analyzed with FlowJo software (Tree Star, Ashland, Ore).
Instrument	FACSCelesta (BD Biosciences)
Software	Flow cytometry data was analyzed by Flowjo v10.<br>Data analysis and plotted were using GraphPad Prism 8.<br>BD DIVA8.0.1 software (BD Biosciences)

Cell population abundance	The abundance is depended on the specific population,from 0-80%.
Gating strategy	All the gating strategy for each experiment was supplied in supplementary figures. They were determined by single color stains where applicable. Generally, the first gating (FSC/SSC) was done on alive cells to exclude debris and dead cells, followed by the section of single cells (FSC-W/FSC-H), excluding clumps. Then the specific fluorophores were confirmed and analyzed by comparing single color stains.

☒ Tick this box to confirm that a figure exemplifying the gating strategy is provided in the Supplementary Information.
